# Qualifying the T-2 Toxin-Degrading Properties of Seven Microbes with Zebrafish Embryo Microinjection Method

**DOI:** 10.3390/toxins12070460

**Published:** 2020-07-18

**Authors:** Edina Garai, Anita Risa, Emese Varga, Mátyás Cserháti, Balázs Kriszt, Béla Urbányi, Zsolt Csenki

**Affiliations:** 1 Department of Aquaculture, Institute for Conservation of Natural Resources, Faculty of Agricultural and Environmental Sciences, Szent István University, H-2100 Gödöllő, Hungary; edina.garai@phd.uni-szie.hu (E.G.); urbanyi.bela@mkk.szie.hu (B.U.); 2 Department of Environmental Safety and Ecotoxicology, Institute for Conservation of Natural Resources, Faculty of Agricultural and Environmental Sciences, Szent István University, H-2100 Gödöllő, Hungary; ranita513@gmail.com (A.R.); cserhati.matyas@mkk.szie.hu (M.C.); kriszt.balazs@mkk.szie.hu (B.K.); 3 Department of Applied Chemistry, Faculty of Food Science, Szent István University, H-2100 Gödöllő, Hungary; varga.emese@etk.szie.hu

**Keywords:** Trichothecene, biodegradation, metabolites, phenotype, *Rhodococcus erythropolis*

## Abstract

T-2 mycotoxin degradation and detoxification efficiency of seven bacterial strains were investigated with zebrafish microinjection method in three steps ((1) determination of mycotoxin toxicity baseline, (2) examination of bacterial metabolites toxicity, (3) identification of degradation products toxicity). Toxicity of T-2 was used as a baseline of toxic effects, bacterial metabolites of strains as control of bacterial toxicity and degradation products of toxin as control of biodegradation were injected into one-cell stage embryos in the same experiment. The results of in vivo tests were checked and supplemented with UHPLC-MS/MS measurement of T-2 concentration of samples. Results showed that the *Rhodococcus erythropolis* NI1 strain was the only one of the seven tested (*R. gordoniae* AK38, *R. ruber* N361, *R. coprophilus* N774, *R. rhodochrous* NI2, *R. globerulus* N58, *Gordonia paraffinivorans* NZS14), which was appropriated to criteria all aspects (bacterial and degradation metabolites of strains caused lower toxicity effects than T-2, and strains were able to degrade T-2 mycotoxin). Bacterial and degradation metabolites of the NI1 strain caused slight lethal and sublethal effects on zebrafish embryos at 72- and 120-h postinjection. Results demonstrated that the three-step zebrafish microinjection method is well-suited to the determination and classification of different bacterial strains by their mycotoxin degradation and detoxification efficiency.

## 1. Introduction

Mycotoxins are global food and feed chain pollutants. In recent years, climate change has increased the size of the areas suitable for fungal growth [1,2]. These secondary metabolites of fungi cause different toxic effects and considerable economic loss [1,3,4].

Trichothecenes are one of the largest mycotoxin group, produced by various species of *Fusarium*, *Myrothecium*, *Trichoderma*, *Trichothecium*, *Cephalosporium*, *Verticimonosporium* and *Stachyobotrys* [5]. Trichothecenes have low molecular weight, the same basic ring structure and a typical 12,13-epoxide group [5,6,7]. The main exposure risks are wheat, barley and maize and other potential ways to enter food chains such as through milk, meat and eggs [5].

T-2 was one of the first discovered mycotoxins, and this toxin is the most toxic among trichothecenes [8]. T-2 has several ways of exposure (oral, parental, dermal and aerosol), which have different targets. In general, acute toxicological effects are feed refusal, dermatitis, vomiting, hemorrhages and necrosis of stomach, testis and ovary aberrations in cats, dairy cattle, dogs, pigs and ducklings [9,10,11,12]. Chronic exposure causes excessive salivation, dizziness, fatigue, abdominal pain and secondary infections (pneumonia) in chickens [13], mice [14], rats [15] and rhesus monkeys [16]. Similar effects have been reported in fish, such as depressed growth, the efficiency of use of feed in young rainbow trout [17], and early life stage toxicity test on zebrafish embryos was conducted [18]. The effects on humans could be connected to Alimentary Toxic Aleukia (ATA) [11,12,19,20,21]. However, it is not clear whether T-2 causes the disease alone or with other mycotoxins [11,12]. ATA is characterized by agranulocytosis, sepsis, atrophy of the bone marrow and mortality. Incidences of the disease were in the USSR (1941–1947), China (1984–1985) and India (1987) [11,12,19,20,21].

These health risks justify the elimination of T-2 toxin from the food and feed chain. Removal and detoxification of mycotoxins by several tools have been investigated [6,20,22]. The first option is preventing the development of mold in the fields, such as the selection of resistant crop varieties [23], suitable cultivation techniques [24], avoiding damage from pests [25] and biocontrol methods [26,27]. This option is often not sufficient without other methods [28]. The second possibilities are postinfection strategies, like physical methods, chemical transformation and biological degradation [29,30,31]. Physical methods, however, are not able to degrade or remove all toxins [5,32,33], while the chemical transformation of mycotoxins decreases nutritional values and produces toxic derivatives [5,34,35,36].

Biological transformation is probably the best available option to decrease mycotoxins. Bacterial degradation of T-2 was described with different strains in many previous studies, such as *Lactobacillus* [37], *Rhodococcus* [38], *Pseudomonas*, *Artrobacter-Brevibacterium* [39], *Curtobacterium* [40], BBSH 797 strains [7]. However, more toxic metabolites have been described in the transformation of T-2, such as 3′-hydroxy-T-2, 3′-hydroxy-HT-2 and 4′-hydroxy-T-2 [39,41,42,43]. In addition, mycotoxins are transformed into more or less toxic products, and bacterial metabolites of bacteria could be toxic thus, degradation does not mean detoxification in every case. Therefore, EFSA recommends that the degradation products need to be tested with in vivo toxicological methods [44,45]. Previously, a microinjection-based test was developed for the qualification of degrading bacteria, based on Ochratoxin-A degradation efficiency of *Cupriavidus basilensis* ŐR16 bacteria. This method uses microinjection to introduce Ochratoxin-A, ŐR16 primary and degradation metabolites at different volumes into newly fertilized zebrafish eggs and examine the lethal and sublethal effects on embryos at 72- and 120-h postinjection. Based on the results of this article, this method could be used for the classification of more bacterial strains by their degradation and detoxification capacity [46].

In this study, we investigated whether the microinjection-based method is appropriate for classifying the T-2 biodegradation and detoxification efficiency of seven different bacterial strains. The objective of this experiment was to examine whether these outcomes agreed with analytical measurements and whether degradation metabolites could be specified from the results. Additionally, this study explored which bacteria are suitable to use for the detoxification of T-2-contaminated feed in confined places.

## 2. Results

### 2.1. Effects of T-2 on Injected Zebrafish Embryos

**Figure 1:**
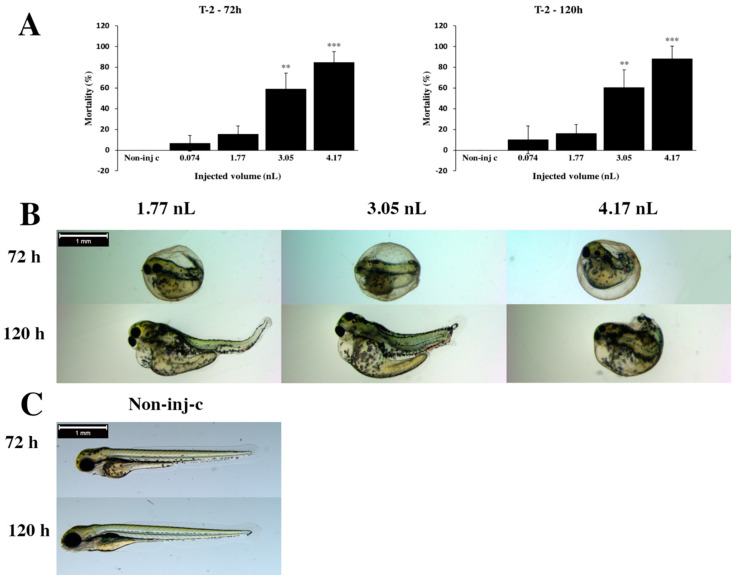
Effects of T-2 mycotoxin injected in different volumes on 72 and 120 hpf zebrafish embryos. Mortality results of T-2 were checked following 72 and 120 h of injection (**A**). Lethality data are expressed as mean ± SD from three independent experiments in triplicate. Kruskal–Wallis followed by Dunn’s post hoc test was used. Mortality values of injected volume were compared to results of noninjected control (** *p* < 0.01, *** *p* < 0.001). T-2 induced representative development dysfunctions in zebrafish embryos following injection were examined after 72 and 120 h of injection (**B**). Typically, hook-like tail, pericardial edema, yolk edema, eye lens, head distortion and lack of hatching were observed. Phenotypes of treated groups were compared to noninjected control (Non-inj-c) groups (**C**). Scale bar: 1 mm. Quantitative data of developmental dysfunctions in zebrafish embryos are in supplementary materials Tables S1 and S2).

The initial concentration of T-2 was 7 mg/L, which was injected in different volumes (0.074, 1.77, 3.05 and 4.17 nL). This T-2 concentration (7 mg/L) was in the concentration range which was not toxic to the bacterial strains in laboratory conditions. Dose–response relationships were observed between mortality and injected volumes at 72 and 120 hpf (hour postfertilization) (Figure 1A). Mortality values increased with each injected volume at longer exposure (120 hpf). At the lowest injected volume (0.074 nL), lethality was 6.67% (±7.64%) at 72 h, which increased to 10% (±13.23%) at 120 hpf. At the maximum (4.17 nL) dose, mortality was 84.6% (±10.64%) at 72 h and 88.0% (±12.55%) at 120 hpf. Significant differences were detected between the noninjected control (Non-inj-c) and the two highest doses (3.05 nL – *p* < 0.01, 4.17 nL – *p* < 0.001) at 72 and 120 h.

Regarding the sublethal effects of T-2, generally, the severity of phenotypic lesions depended on the volume of injected doses (Figure 1B). Lower injected volumes caused significantly less serious sublethal effects (1.77 nL *p* < 0.05, 4.17 nL *p* < 0.01) at 72 and 120 hpf (Tables S1 and S2). Typically, hook-like tail, pericardial edema, yolk edema, eye lens, head distortion and lack of hatching were observed. Lack of hatching was noticed at all injected volumes at 72 h, and it was seen in the largest dose (4.17 nL) at 120 hpf. Phenotypes of treated groups were compared to noninjected control groups (Figure 1C).

### 2.2. Effects of Bacterial Metabolites and Degradation Products on Injected Embryos

Biodegradation and detoxification efficiency of seven different bacterial strains were tested with the microinjection-based method. Toxic effects of bacteria were tested with two types of their products (bacterial and degradation metabolites) with the same injected volumes (0.074, 1.77, 3.05 and 4.17 nL).

**Figure 2:**
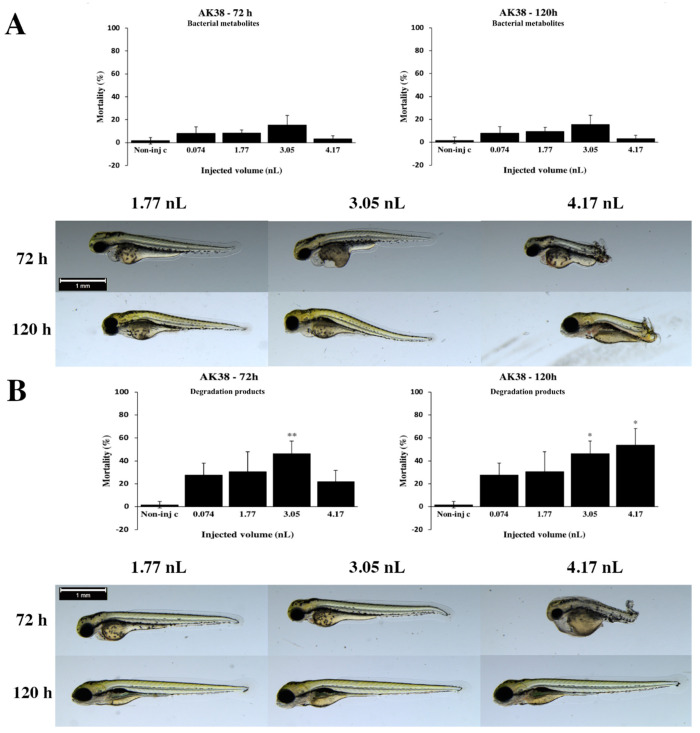
Effects of the *Rhodococcus gordoniae* AK38 strain bacterial (**A**) and degradation (**B**) metabolites injected in different volumes on 72 and 120 hpf zebrafish embryos. Mortality results of the AK38 strain bacterial (**A**, above) and degradation (**B**, above) metabolites were checked following 72 and 120 h of injection. Lethality data are expressed as mean ± SD from three independent experiments in triplicate. Kruskal–Wallis followed by Dunn’s post hoc test was used. Mortality values of injected volume were compared to results of noninjected control (* *p* < 0.05, ** *p* < 0.01). The AK38 strain bacterial (**A**, bottom) and degradation (**B**, bottom) metabolites induced representative development dysfunctions in zebrafish embryos following injection were examined after 72 and 120 h of injection. Typically, tail deformities, pericardial and yolk edema were detected. Scale bar: 1 mm. Phenotypes of treated groups were compared to noninjected control (Non-inj-c) groups (Figure 1C). Quantitative data of developmental dysfunctions in zebrafish embryos are in supplementary materials Tables S1 and S2).

Lethality rates of degradation products of the *Rhodococcus gordoniae* AK38 strain increased with the injected volume of 3.05 nL at 72 h (Figure 2B). At 120 h, mortality results showed a clear dose–response relationship. Statistically significant differences were detected between noninjected control and 3.05 nL (*p* < 0.01) at 72 h, noninjected control and 3.05 nL (*p* < 0.05) and 4.17 nL (*p* < 0.05) at 120 hpf. In general, the severity of sublethal effects of degradation products depended on the volume of injected doses (Figure 2B). Lower injected volumes caused significantly less serious tail deformities (3.05 nL *p* < 0.05, 4.17 nL *p* < 0.05) at 72 h (Tables S1 and S2). Typically, tail deformities, pericardial and yolk edema were detected at 72 hpf. At 120 h, swim bladders of larvae were not developed.

**Figure 3:**
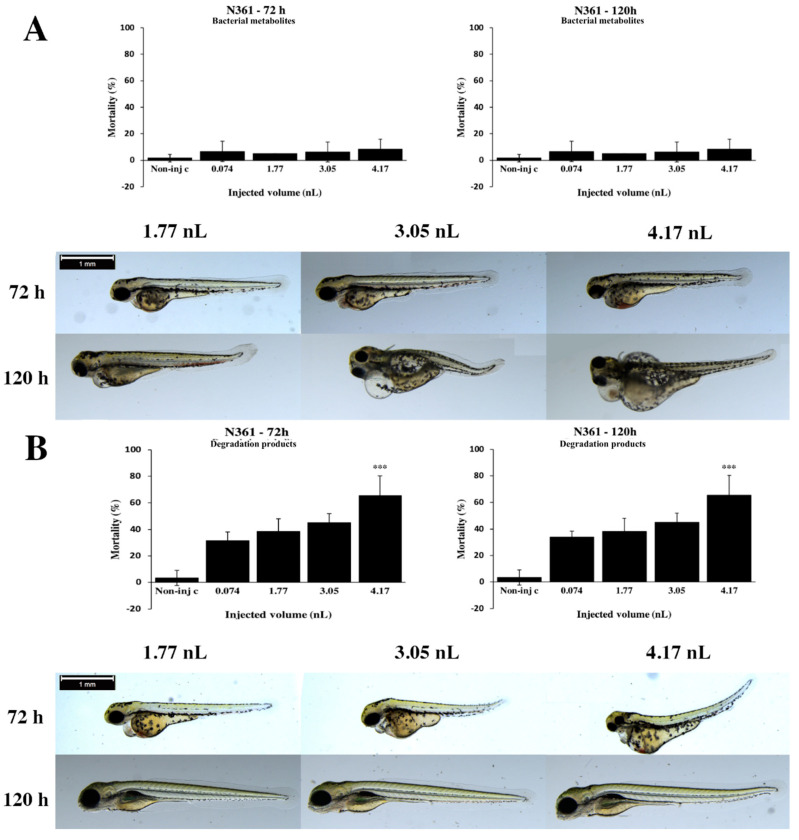
Effects of the *Rhodococcus ruber* N361 strain bacterial (**A**) and degradation (**B**) metabolites injected in different volumes on 72 and 120 hpf zebrafish embryos. Mortality results of the N361 strain bacterial (**A**, above) and degradation (**B**, above) metabolites were checked following 72 and 120 h of injection. Lethality data are expressed as mean ± SD from three independent experiments in triplicate. Kruskal–Wallis followed by Dunn’s post hoc test was used. Mortality values of injected volume were compared to the results of noninjected control (** *p* < 0.01). The N361 strain bacterial (**A**, bottom) and degradation (**B**, bottom) metabolites induced representative development dysfunctions in zebrafish embryos following injection were examined after 72 and 120 h of injection. Typically, tail deformities, pericardial and yolk edema and head distortion were detected. Scale bar: 1 mm. Phenotypes of treated groups were compared to noninjected control (Non-inj-c) groups (Figure 1C). Quantitative data of developmental dysfunctions in zebrafish embryos are in supplementary materials Tables S1 and S2).

Mortality results of degradation products of the *Rhodococcus ruber* N361 strain have shown a similar dose–response relationship at 72 and 120 h (Figure 3B). The maximum mortality was 65.63% (±14.77%) at 4.17 nL dose at 72 and 120 hpf. Significant differences were detected between the noninjected control and 4.17 nL (*p* < 0.001) at 72 and 120 hpf. Ordinarily, the severity of phenotypic lesions of degradation products depended on the volume of injected doses (Figure 3B). Smaller injected volumes caused significantly less serious tail deformities, pericardial and yolk edema (3.05 nL *p* < 0.05, 4.17 nL *p* < 0.01) at 72 hpf. Typically, tail deformities, pericardial and yolk edema and head distortion were detected at 72 hpf. Larger injected volumes caused significantly more severe tail deformities (3.05 nL *p* < 0.05, 4.17 nL *p* < 0.01) at 120 h (Tables S1 and S2).

**Figure 4:**
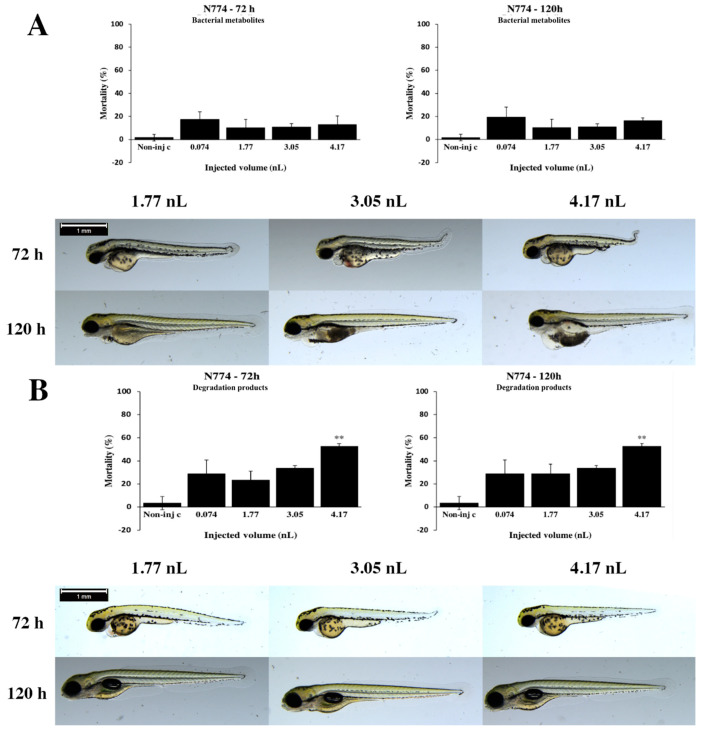
Effects of the *Rhodococcus coprophilus* N774 strain bacterial (**A**) and degradation (**B**) metabolites injected in different volumes on 72 and 120 hpf zebrafish embryos. Mortality results of the N774 strain bacterial (**A**, above) and degradation (**B**, above) metabolites were checked following 72 and 120 h of injection. Lethality data are expressed as mean ± SD from three independent experiments in triplicate. Kruskal–Wallis followed by Dunn’s post hoc test was used. Mortality values of injected volume were compared to the results of noninjected control (** *p* < 0.01). The N774 strain bacterial (**A**, bottom) and degradation (**B**, bottom) metabolites induced representative development dysfunctions in zebrafish embryos following injection were examined after 72 and 120 h of injection. Representative phenotypic lesions were tail deformities, pericardial edema, head and lens distortion and yolk edema. Scale bar: 1 mm. Phenotypes of treated groups were compared to noninjected control (Non-inj-c) groups (Figure 1C). Quantitative data of developmental dysfunctions in zebrafish embryos are in supplementary materials Tables S1 and S2).

Phenotypic lesions caused by bacterial metabolites of the *Rhodococcus coprophilus* N774 strain depended on the volume of injected doses (Figure 4A). Lower injected volumes caused significantly less serious tail deformities (4.17 nL *p* < 0.05) at 72 hpf (Tables S1 and S2). Larger injected volumes caused significantly more severe yolk deformed (4.17 nL *p* < 0.05) at 120 h (Tables S1 and S2). Representative phenotypic lesions were tail deformities pericardial edema, head and lens distortion and yolk edema at 72 and 120 hpf. Degradation products of the N774 strain lethality results were shown dose–response relationship (Figure 4B). The maximum mortality result was 52.54% (±2.5%) at 4.17 nL dose. Statistically significant differences were detected between noninjected control and 4.17 nL (*p* < 0.01) at 72 and 120 hpf. Commonly, the severity of sublethal effects of degradation products depended on the volume of injected doses (Figure 4B). Smaller injected volumes caused significantly less serious yolk and pericardial edema (3.05 nL *p* < 0.05, 4.17 nL *p* < 0.01) at 72 hpf (Tables S1 and S2). Typically, tail deformities, pericardial and yolk edema and head distortion were detected at 72 hpf. At 120 h, larvae were not phenotypic lesions at either dose.

**Figure 5:**
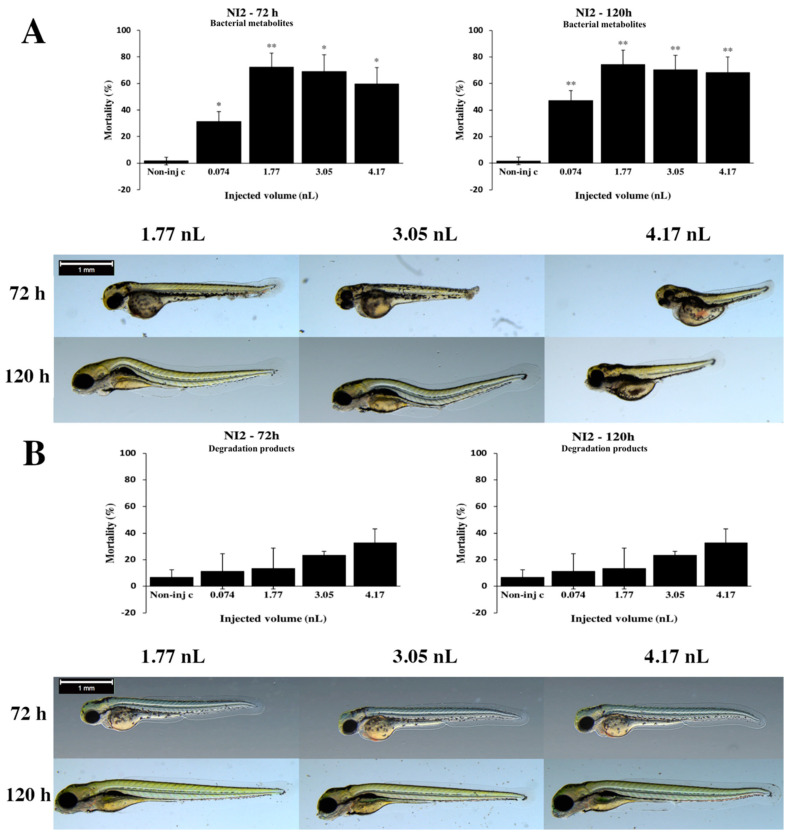
Effects of the *Rhodococcus rhodochrous* NI2 strain bacterial (**A**) and degradation (**B**) metabolites injected in different volumes on 72 and 120 hpf zebrafish embryos. Mortality results of the NI2 strain bacterial (**A**, above) and degradation (**B**, above) metabolites were checked following 72 and 120 h of injection. Lethality data are expressed as mean ± SD from three independent experiments in triplicate. Kruskal–Wallis followed by Dunn’s post hoc test was used. Mortality values of injected volume were compared to results of noninjected control (* *p* < 0.05, ** *p* < 0.01). The NI2 strain bacterial (**A**, bottom) and degradation (**B**, bottom) metabolites induced representative development dysfunctions in zebrafish embryos following injection were examined after 72 and 120 h of injection. Representative phenotypic lesions were tail deformities, pericardial edema, head and lens distortion and yolk edema. Scale bar: 1 mm. Phenotypes of treated groups were compared to noninjected control (Non-inj-c) groups (Figure 1C). Quantitative data of developmental dysfunctions in zebrafish embryos are in supplementary materials Tables S1 and S2).

Bacterial metabolites of the *Rhodococcus rhodochrous* NI2 lethality values increased to 120 h at each injected volume (Figure 5A). The maximum mortality value was 72.14% (±10.59%) at 1.77 nL dose at 72 hpf. Statistically significant differences were observed between noninjected control and all injected doses (0.074 nL, 3.05 nL and 4.17 nL: *p* < 0.05, 1.77 nL: *p* < 0.01) at 72 h. At 120 h, the maximum lethality value was 74.14% (±11.02%) at 1.77 nL. Statistically significant differences were detected between noninjected control and all injected doses (*p* < 0.01) at 120 hpf. In general, the seriousness of phenotypic distortion of bacterial metabolites depended on the volume of injected doses (Figure 5A). Larger injected volumes caused significantly more severe tail deformities and yolk edema (3.05 nL *p* < 0.01, 4.17 nL *p* < 0.001) at 72 and 120 hpf (Tables S1 and S2). Representative phenotypic lesions were tail deformities, pericardial edema, head and lens distortion and yolk edema at 72 and 120 hpf. Degradation products of the NI2 strain phenotypic lesions depended on the volume of injected doses (Figure 5B). Smaller injected volumes caused significantly less serious tail deformities (4.17 nL *p* < 0.05) at 72 and 120 hpf (Tables S1 and S2). Typically, tail deformities, pericardial edema and head distortion were detected at 72 and 120 hpf.

**Figure 6:**
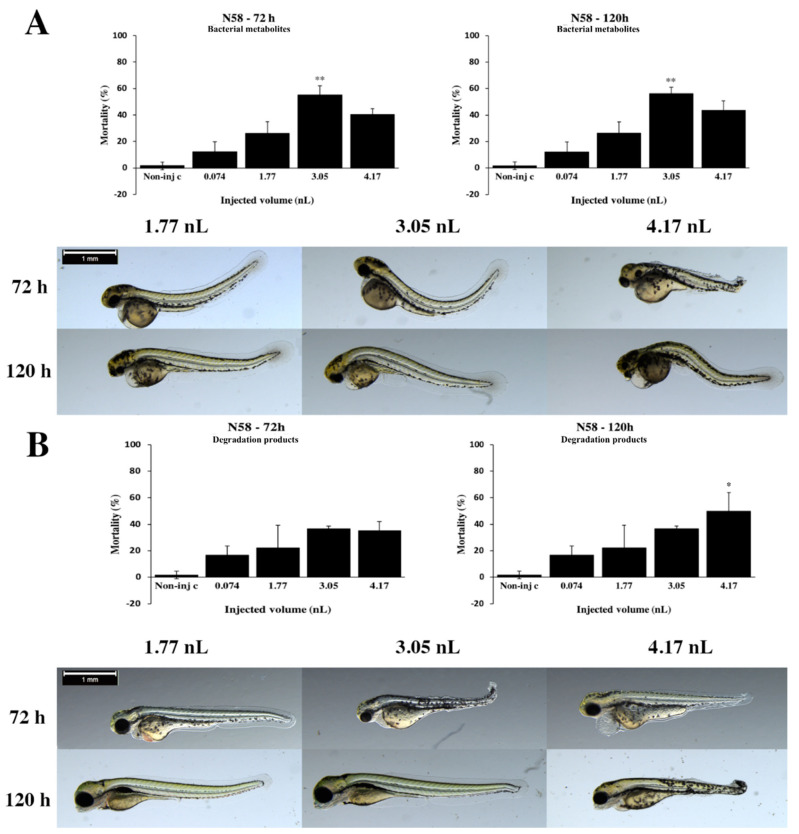
Effects of the *Rhodococcus globerulus* N58 strain bacterial (**A**) and degradation (**B**) metabolites injected in different volumes on 72 and 120 hpf zebrafish embryos. Mortality results of the N58 strain bacterial (**A**, above) and degradation (**B**, above) metabolites were checked following 72 and 120 h of injection. Lethality data are expressed as mean ± SD from three independent experiments in triplicate. Kruskal–Wallis followed by Dunn’s post hoc test was used. Mortality values of injected volume were compared to the results of noninjected control (** *p* < 0.01). The N58 strain bacterial (**A**, bottom) and degradation (**B**, bottom) metabolites induced representative development dysfunctions in zebrafish embryos following injection were examined after 72 and 120 h of injection. Typically, yolk edema, tail deformities, pericardial edema and head and lens distortion were detected. Scale bar: 1 mm. Phenotypes of treated groups were compared to noninjected control (Non-inj-c) groups (Figure 1C). Quantitative data of developmental dysfunctions in zebrafish embryos are in supplementary materials Tables S1 and S2).

Bacterial metabolites of the *Rhodococcus globerulus* N58 strain mortality rates increased with the injected volume to 3.05 nL at 72 and 120 h (Figure 6A). The maximum mortality value was 56.25% (±4.79%) at a 3.05 nL dose at 120 hpf. Statistically significant differences were observed between noninjected control and 3.05 nL (*p* < 0.01) at 72 and 120 h. Ordinarily, the severity of sublethal effects of bacterial metabolites depended on the volume of injected doses (Figure 6A). Larger injected volumes caused significantly more severe tail deformities, pericardial, yolk edema and head distortion (4.17 nL *p* < 0.05) at 72 and 120 hpf (Tables S1 and S2). Representative phenotypic lesions were tail deformities, pericardial edema, head and lens distortion and yolk edema at 72 and 120 hpf. Degradation products of the N58 strain lethality results were shown to have a dose–response relationship at 120 h (Figure 6B). Mortality values were not changed to 120 h except for 4.17 nL, which was increased from 35% (±7.07%) to 50% (±14.14%). Statistically significant differences were detected between noninjected control and 4.17 nL at 120 hpf. Generally, the seriousness of phenotypic lesions of degradation products depended on the volume of injected doses (Figure 6B). Smaller injected volumes caused significantly less serious tail deformities, pericardial, yolk edema and head distortion (4.17 nL, *p* < 0.01) at 72 and 120 hpf (Tables S1 and S2). Typically, yolk edema, tail deformities, pericardial edema and head and lens distortion were detected at 72 and 120 hpf.

**Figure 7:**
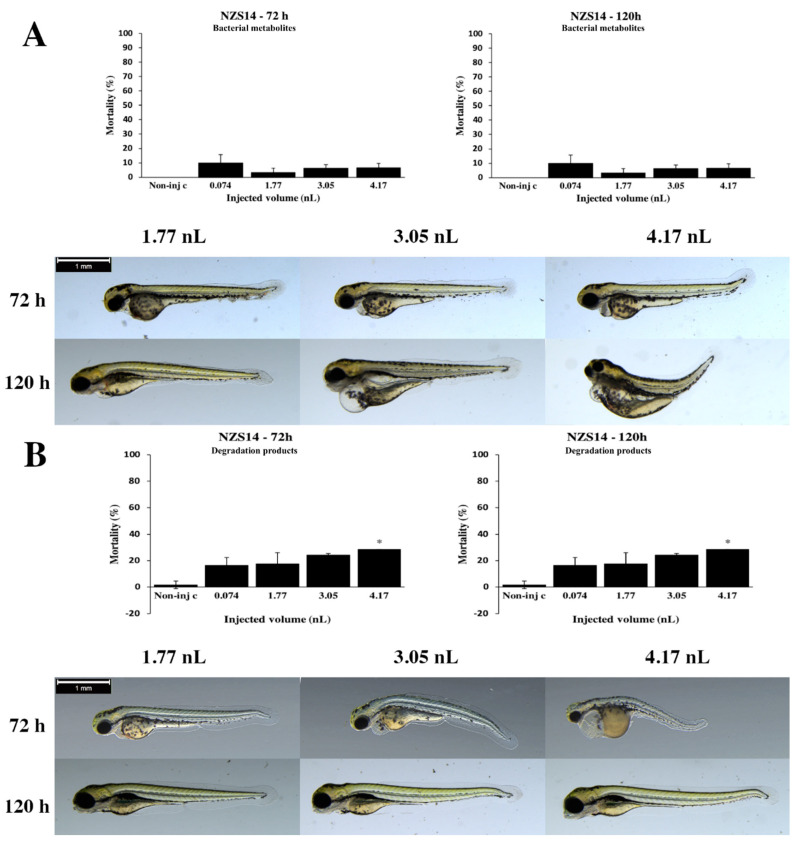
Effects of the *Gordonia paraffinivorans* NZS14 strain bacterial (**A**) and degradation (**B**) metabolites injected in different volumes on 72 and 120 hpf zebrafish embryos. Mortality results of the NZS14 strain bacterial (**A**, above) and degradation (**B**, above) metabolites were checked following 72 and 120 h of injection. Lethality data are expressed as mean ± SD from three independent experiments in triplicate. Kruskal–Wallis followed by Dunn’s post hoc test was used. Mortality values of injected volume were compared to results of noninjected control (* *p* < 0.05). The NZS14 strain bacterial (**A**, bottom) and degradation (**B**, bottom) metabolites induced representative development dysfunctions in zebrafish embryos following injection were examined after 72 and 120 h of injection. Typically, yolk edema, tail deformities, pericardial edema and head and lens distortion were detected. Scale bar: 1 mm. Phenotypes of treated groups were compared to noninjected control (Non-inj-c) groups (Figure 1C). Quantitative data of developmental dysfunctions in zebrafish embryos are in supplementary materials Tables S1 and S2).

Bacterial metabolites of the *Gordonia paraffinivorans* NZS14 strain phenotypic lesions depended on the volume of injected doses (Figure 7A). Larger injected volumes caused significantly more severe tail deformities, pericardial and yolk edema (4.17 nL *p* < 0.05) at 72 and 120 hpf (Tables S1 and S2). Representative phenotypic lesions were tail deformities, pericardial edema, head and lens distortion and yolk edema at 72 and 120 hpf. Degradation products of the NZS14 strain lethality results were shown dose–response relationship (Figure 7B). The maximum mortality rate was 28.57% (±0%) at 4.17 nL at 72 and 120 hpf. Statistically significant differences were detected between noninjected control and 4.17 nL injected volume (*p* < 0.05) at 72 and 120 hpf. In general, the seriousness of phenotypic lesions of degradation products depended on the volume of injected doses (Figure 7B). Smaller injected volumes caused significantly less serious tail deformities, pericardial and yolk edema (4.17 nL *p* < 0.05) at 72 hpf (Tables S1 and S2). Larger injected volumes caused significantly more severe tail deformities and head distortion (4.17 nL *p* < 0.05) at 120 hpf (Tables S1 and S2). Typically, yolk edema, tail deformities, pericardial edema and head and lens distortion were detected at 72 and 120 hpf.

**Figure 8:**
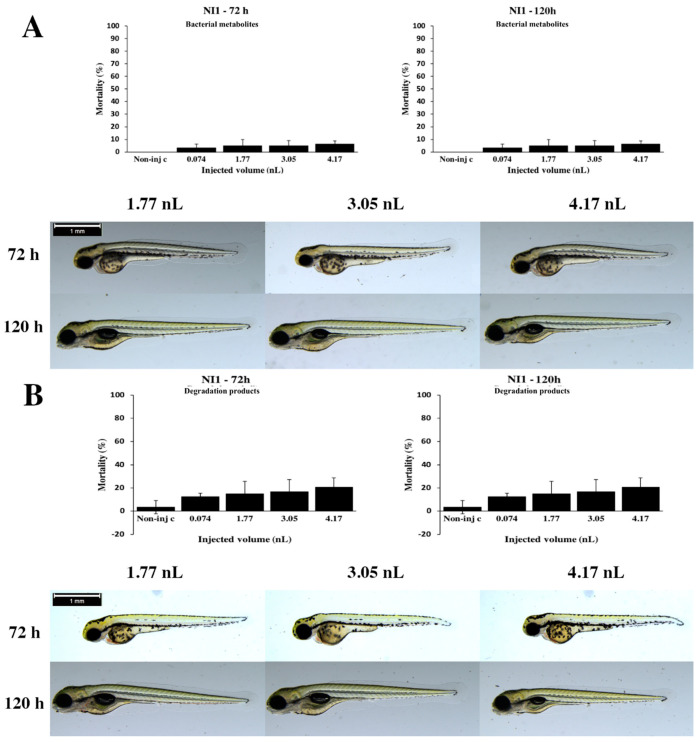
Effects of the *Rhodococcus erythropolis* NI1 strain bacterial (**A**) and degradation (**B**) metabolites injected in different volumes on 72 and 120 hpf zebrafish embryos. Mortality results of the NI1 strain bacterial (**A**, above) and degradation (**B**, above) metabolites were checked following 72 and 120 h of injection. Lethality data are expressed as mean ± SD from three independent experiments in triplicate. Kruskal–Wallis followed by Dunn’s post hoc test was used. Mortality values of injected volume were compared to the results of noninjected control. The NI1 strain bacterial (**A**, bottom) and degradation (**B**, bottom) metabolites induced representative development dysfunctions in zebrafish embryos following injection were examined after 72 and 120 h of injection. Typically, yolk edema, tail deformities and pericardial edema were detected. Scale bar: 1 mm. Phenotypes of treated groups were compared to noninjected control (Non-inj-c) groups (Figure 1C). Quantitative data of developmental dysfunctions in zebrafish embryos are in supplementary materials Tables S1 and S2).

Statistically significant differences were not detected in other results (Figure 8).

### 2.3. Analytical Results

Analysis of T-2 concentration was performed using an UHPLC-MS/MS (ultra-high-performance liquid chromatography hyphenated with tandem mass spectrometer). The supernatants and pellets were evaluated separately (Table 1). AK38 bacterial samples contained a higher volume of T-2 in the supernatant (5.42 ± 0.44 mg/L), than in the pellet (1.73 ± 0.73 mg/L). The AK38 strain was not able to degrade 7 mg/L T-2 toxin in five days. The N774 strain supernatant samples included T-2 (1.39 ± 1.09 mg/L), while in the pellet T-2 concentration was under the detection limit. The N774 bacterial strain was able to degrade almost 80% of mycotoxin. The N58, NI2 and NI1 strains effectively degraded 100% of T-2, their samples (supernatant and pellet) did not contain detectable T-2 levels. N361 samples included T-2, with higher volume in the supernatant (6.29 ± 0.45 mg/L), than in pellet (0.87 ± 0.39 mg/L). The N361 strain was not able to degrade the toxin. NZS14 bacterial supernatant samples contained T-2 (1.45 ± 1.26 mg/L), yet the pellet has no detectable toxin. NZS14 bacterial strain effectively degraded almost 80% of mycotoxin.

## 3. Discussion

The results of the three-step zebrafish microinjection method allowed us to determine which bacteria were able to detoxify and degrade T-2 toxin without toxic effects in the aqueous environment. The first step was the determination of the T-2 toxicity baseline, which had to comply with the criteria described below. The maximum injectable volume for zebrafish embryos is 4.17 nL corresponding to a sphere diameter of 200 µm [46,47]. Concentration and injected volumes of mycotoxin should be selected, so that mortality values are interpretable above and below the baseline in every injected volume. In this study, the experimental design (7 mg/L T-2 concentration and selected injected volumes) corresponded to these criteria. Our results are in agreement with previous studies, which also suggest that T-2 toxin-induced developmental toxicity in zebrafish embryos [18]. We found that the two largest injected doses of T-2 caused significantly higher mortality rates in embryos, 2.71 nL (95% CI (confidence interval): 2.54–2.89 nL) injected dose caused half of the embryos’ mortality. In a previous study, zebrafish embryos were sensitive to the toxicity of T-2 toxin, lethal concentration (LC_50_) was 0.28 µmol/L [18], which was lower than the one in this study. Also, earlier studies have reported that acute toxicity of the T-2 toxin is high in model and domestic animals, LD_50_ (lethal dose): 0.05–1.5 mg/kg bw in rat [48], LD50: 0.4–2 mg/kg bw in Guinea pigs [48], LD_50_: 2.1–10 mg/kg bw (body weight) in mice [49], LD50: 1.1 mg/kg bw in rabbits [50], LD50: 5.03–5.46 mg/kg bw in broilers [51], LD_50_: 1.21 mg/kg bw in pigs [52], LD_50_: 5 mg/kg bw in poultry [51], LD_50_: 0.65 mg/kg bw in monkeys [42]. These results demonstrate that zebrafish is one of the most sensitive vertebrates to T-2 toxin, therefore an ideal model organism for these tests.

Exposure to T-2 also caused phenotypic lesions. The severity of tail deformities, pericardial and yolk edema and lack of swimming behavior increased with injected volumes, which results are in good agreement with a previous study on zebrafish embryos [18]. In addition to these, we found the seriousness of lens and head distortion increased with injected volume, which had not been reported previously. Also, studies have reported the developmental toxicity of T-2 toxin in models and domestic animals, such as abnormal positioning of wings in poultry [53], growth retardation in pigs [54], shortened or missing tails in mice [55], reduced growth rates in broiler chickens [56], delayed growth in sheep [57], depressed growth in rainbow trout [17]. In a previous study on zebrafish embryos, T-2 induced apoptosis in the tail and resulted in the hook-like tail malformation [18], similarly to the findings in the present work. Previous studies have reported T-2-induced apoptosis in animals and human cells [42].

The second step was the examination of the toxicity of bacterial metabolites, which were tested with the same four injected volumes as the ones used for the analysis of mycotoxin baseline. Bacteria are able to produce toxic metabolites under normal living conditions [46]. The effects of these bacterial metabolites need to be examined. If these are toxic, the bacteria should be excluded from the list of potentially environmentally safe microbes in this experiment. We found that bacterial metabolites of the *Rhodococcus globerulus* N58 caused high mortality at the two highest injected doses, and bacterial metabolites of the *Rhodococcus rhodochrous* NI2 strains significantly increased lethality at each injected volume on zebrafish embryos at 72 and 120 h. Therefore, these strains were excluded from the list of environmentally safe microbes. These results are in good agreement with a previous study [58], which also described that bacteria are able to produce toxic metabolites under normal living conditions, such as *Rhodococcus pyridinivorans* CHB15P, *Rhodococcus erythropolis* NI1 and the combination from these strains caused cytotoxicity in *Aliivibrio fischeri* [58]. Contrarily to the data presented here, bacterial metabolites of the *Rhodococcus erythropolis* NI1 strain caused minimal mortality rate in zebrafish embryos (Figure 8A). Additionally, this study explored which bacteria would be environmentally safe in a fish system, and whether bacteria could produce toxic substances without toxic materials. Our results demonstrate that bacterial metabolites of the *Rhodococcus coprophilus* N774 and *Rhodococcus gordoniae* AK38 (Figure 2A) strains caused low lethality and *Rhodococcus ruber* N361 (Figure 3A), *Gordonia paraffinivorans* NZS14 and *Rhodococcus erythropolis* NI1 strains caused minimal mortality at 120 h. Based on the results of bacterial metabolites, these five bacterial strains can be listed as safe microbes in this fish system. Members of the genus *Rhodococcus* can transform a wide range of toxic substances, this metabolic diversity is related to the presence of linear plasmids and large genome sizes, which include large sets of oxidases and other enzymes [38].

The third step was the identification of the toxicity of degradation products, which were tested with the same four injected volumes as mycotoxin baseline were analyzed. Mortality data of degradation products can show whether bacteria are able to degrade mycotoxin, or if the strains produce any toxic degradation products during biodegradation. Earlier studies have reported that T-2 toxin is rapidly metabolized in different species [42,59]. The predominant metabolic transformation is T-2 toxin to HT-2, which is a nontoxic metabolite; and one activation pathway is known, which leads to 3′-hydroxy-T-2, 3′-hydroxy-HT-2, 4′-hydroxy-T-2, which are more toxic than T-2 [39,41,42,43]. Therefore, the biological transformation of T-2 does not mean detoxification in every case. From the data presented, degradation products of the *R. gordoniae* AK38 strain caused significantly high mortality rates in zebrafish embryos at 120 h, which means that the strain was not able to degrade T-2 in five days. The lethality of AK38 degradation products did not reach the toxicity rates of T-2 thus, it can be concluded that bacteria are probably able to bind T-2 toxin [38]. Previous studies suggest that different bacteria are able to bind the mycotoxin due to the structure of the cell walls [37]. These outcomes were in agreement with analytical results, which showed that the supernatants and pellets from the AK38 strain contained all added T-2 toxins. Contrary to our results, an earlier study found that the AK38 strain was able to degrade or bind 90% of T-2 mycotoxin [38]. However in the above-mentioned experiment, the concentration of mycotoxin was 2 mg/L compared to 7 mg/L used in our study, and the test duration was three days compared to five days; finally, the most important difference between the two studies was the concentration of LB (Luria-Bertani) medium, which was 100% in the earlier test and 20% in this study [38]. In this work, the concentration of the LB medium was 20% during degradation experiments in order for the bacteria to use T-2 toxin as a primary source of carbon. It can be concluded that the AK38 strain requires a 100% LB medium for T-2 toxin degradation. Based on these, a future study of the AK38 strain would likely provide more information in terms of the ability to bind or degrade T-2 toxin.

Our results demonstrate that the degradation products of the *R. globerulus* N58 strain caused the same rate of lethality than bacterial metabolites. The phenotypic lesions are also completely the same, severity of tail deformities, pericardial and yolk edema as well as head distortion increased with the injected dose at 72 and 120 h. Therefore, this strain was able to degrade 100% of T-2 toxin and bacterial metabolites generated lethality and deformity in zebrafish embryos. These results were in agreement with the analytical results, which showed that neither the supernatants nor the pellets from N58 did not contain T-2 toxin in detectable concentration. Analytical results are in good agreement with previously published results [38].

From the data presented, degradation products of the *R. ruber* N361 strain caused a significantly higher mortality rate on zebrafish embryos at 120 h, which means the strain was not able to degrade T-2 in five days. The lethality of N361 degradation products did not reach the rates of T-2 thus, it can be concluded that these bacteria are probably able to bind T-2 toxin, similarly to the AK38 strain. These outcomes were in agreement with analytical results, which showed that the supernatants and pellets from the N361 strain contained all added T-2 toxin. Contrary to our results, an earlier study found that the AK38 strain was able to degrade 60% of T-2 mycotoxins [38]. This difference can be for the same reasons as that described with the AK38 strain.

Our results demonstrate that degradation products of the *R. coprophilus* N774 strain caused high mortality in zebrafish embryos at 120 h, which rate was not high as T-2 lethality. Therefore, the strain was not able to degrade all added T-2 toxin in five days. Analytical results showed that bacteria were able to degrade almost 80% of T-2 and the supernatants from the N774 strain contained T-2 in low volumes. It can be concluded that N774 bacterial strain probably degrades the T-2 on the activation pathway and 3′-hydroxy-T-2, 3′-hydroxy-HT-2 or 4′-hydroxy-T-2 metabolites are formed. Earlier studies have reported that these T-2 metabolites caused toxicity in rats and rat hepatic S-9 preparations and were found in the urine of lactating cow and swine, as well as in chicken excreta and tissues [43]. Contrary to our analytical measurements, an earlier study has found that the N774 strain was able to degrade almost 100% of T-2 mycotoxins, which difference can be attributed to the same reasons as AK38 and N361 strains [38]. From the data presented, degradation products of the *Gordonia paraffinivorans* NZS14 strain caused intermediate mortality rates in zebrafish embryos at 120 h, which means that the bacteria were not able to degrade all added T-2 toxin in five days. These results are in agreement with analytical results, which showed that bacteria were able to degrade almost 80% of T-2 and the supernatants from the NZS14 strain contained T-2 in low amounts.

Our outcomes demonstrate that the NI2 strain was able to degrade 100% T-2 in five days, based on mortality and sublethal results of degradation products. The maximum lethality rate of NI2 degradation products did not reach the T-2 and bacterial metabolite values, it can be concluded that bacteria probably use different types of genes for normal life processes and to degrade toxins. Previous studies suggest that in the transcriptome, there are relevant changes between mycotoxin conditions and control. The biological responses of bacteria in the presence of mycotoxins are detected with RNA-seq technology. Escherichia coli K-12 genes, which are most differentially expressed, are downregulated in response to deoxynivalenol and nivalenol, which toxins also belong to the group of trichothecenes [60]. These results correspond to analytical measurements, which show that neither the supernatants nor the pellets from NI2 contain the T-2 toxin in detectable concentration. Our analytical data are in good agreement with those published earlier [38].

We found that *R. erythropolis* NI1 bacteria were able to degrade 100% of mycotoxin based on slight mortality and sublethal results of degradation products. Data can be concluded that T-2 degradation products are not toxic, and slight mortality is probably caused by bacterial metabolites of the NI1 strain (Figure 8B). These results are in agreement with analytical results, which showed that neither the supernatants nor the pellets from NI1 contained T-2 toxin in detectable concentration. Our analytical data are in good agreement with those published earlier [38]. From the data presented, the three-step microinjection-based method is well-suited to the rank of seven T-2 degradable bacterial strains, based on their efficiency of degradation and detoxification, and furthermore, it is well-suited to select of bacteria, which can detoxify and degrade T-2 toxin without toxic effects in fish systems. The results of this study demonstrate that the *Rhodococcus erythropolis* NI1 bacterial strain is appropriate for these criteria, and it would be suitable to use for the detoxification of T-2-contaminated feed in confined places.

## 4. Conclusions

The microinjection-based method with three steps experimental design ((1) determination of mycotoxin toxicity baseline, (2) examination of bacterial metabolites toxicity, (3) identification of degradation products toxicity) is a quick and accurate qualification process of bacterial degradation efficiency. This in vivo tool is appropriate to rank other mycotoxins or xenobiotics and bacteria that are able to degrade them in case of degradation of the same toxic substance. This was proven by examining the efficiency of seven bacterial strains to degrade T-2 toxin. The method may offer a general in vivo test option that can provide more information even without analytical tests.

The results demonstrated that the *Rhodococcus globerulus* N58 and *Rhodococcus rhodochrous* NI2 strains produce toxic bacterial metabolites, *Rhodococcus coprophilus* N774 and *Gordonia paraffinivorans* NZS14 strains produce toxic degradation metabolites in zebrafish embryos, *Rhodococcus gordoniae* AK38 and *Rhodococcus ruber* N361 strains were not able to degrade T-2 toxin; thus, the strains were not appropriate because of these. The results of this study demonstrate that the *Rhodococcus erythropolis* NI1 bacterial strain is the only one of the seven tested, which is appropriate for the criteria (bacterial and degradation metabolites of strains caused lower toxicity effects than T-2, and strains were able to degraded T-2 mycotoxin), it would be suitable to use for the detoxification of T-2-contaminated feed in confined places. The application of microbial detoxification agents in the food and feed chain seems to be decreasing, because of toxicological and consumer considerations, and regulations. Therefore, future studies should concentrate on the determination of genes, which are responsible for T-2 biodegradation as these genes would be better alternate methods to eliminate T-2 toxin.

## 5. Material and Methods

### 5.1. Animal Protection

The Animal Protocol (2013) was approved under the Hungarian Government Regulation on animal experiments (42/2013. (II.4.)) and all studies were completed before the treated individuals would have reached the free-feeding stage.

### 5.2. Bacterial Experiments

Seven bacterial strains were selected by their degradation efficiency from the strain collection of our institute (Institute for Conservation of Natural Resources). We selected strains with high degradation efficiency, in order to also determine their detoxification efficiency also.

Seven bacterial strains (stored at −80 °C) were streaked on LB agar plates (10 g tryptone, 5 g yeast extract, 9 g sodium-chloride and 18 g bacteriological agar (Biolab Ltd., Budapest, Hungary) in 1L (pH 7.0) ion-exchanged water) and incubated at 28 °C for 72 h. Then single colonies from each strain were inoculated into 50 mL 100% LB medium (10 g tryptone, 5 g yeast extract and 9 g sodium-chloride in 1L (pH 7.0) ion-exchanged water) in 250 mL flasks and cultures were grown for 120 h at 28 °C, 170 rpm in a shaking incubator (Sartorius Certomat BS-1, Göttingen, Germany). Liquid cultures were centrifuged at 3220× *g*, 4 °C for 20 min (Eppendorf 5810R, Hamburg, Germany), the pellets were resuspended in 50 mL 20% LB medium (100% LB medium diluted with ion-exchanged water), then were centrifuged again at the same conditions. The procedure was repeated twice. After resuspension, the optical density of the cultures was measured at 600 nm (OD600) (GENESIS 10S UV-VIS, Thermo Fisher Scientific, MA, USA) and adjusted to 0.6 ± 0.05 to prepare bacterial inoculum. All bacterial experiments were prepared in the same method. A 5 mL volume of bacterial suspensions were inoculated into 45 mL sterile 20% LB medium to test the effects of primary metabolites. Similar inocula were prepared in parallel, which contained T-2 mycotoxin (7 mg/L final concentration (Fermentek Ltd., Israel)). The negative control was an uninoculated 20% LB medium contaminated by T-2 (7 mg/L final concentration). Experiments were incubated on a laboratory shaker at 28 °C, 170 rpm for 120 h in triplicates. Cultures were centrifuged at 3220× *g*, 4°C, for 20 min. Supernatants for microinjection (1 mL) were filtered with 0.2 µm syringe filters (VWR International Ltd., Budapest, Hungary) to gain bacteriologically sterile samples. Pellets and supernatants were stored separately at −20 °C until analytical measurements.

### 5.3. Analytical Measurement

For the measurement of T-2 concentrations, UHPLC-MS/MS (ultra-high-performance liquid chromatography hyphenated with tandem mass spectrometer) was applied. Firstly, pellets were extracted with acetonitrile/water/formic acid (79/20/1, *v*/*v* %) mixture than an aliquot of 500 µL of the extracts were taken into 1.5 mL dark vials. Supernatants were taken directly from an aliquot of 500 µL into 1.5 mL dark vials. Afterwards, both sample types (LB broth and pellet) were evaporated until dryness under a gentle N2 stream. The residues were reconstituted in 50:50 *v*/*v* % A:B mobile phases (A: water, 5 mM ammonium formate, 0.1% formic acid; B: methanol, 5 mM ammonium formate, 0.1% formic acid) and were filtered through an 0.22 µm PTFE (polytetrafluoroethylene) filter. Agilent 1290 Infinity II UHPLC system (Agilent Technologies, Santa Clara, CA 95051, United States) equipped with Agilent Zorbax Eclipse Plus chromatographic column (2.1 × 50 mm, 1.8 μm) was used. A 5 μL volume of prepared samples were injected into the mobile phase, which initially contained 95% A and 5% B eluents. Then, a 400 μL/min flow rate and 40 °C column temperature were set. A triple-quadrupole mass spectrometer (Ultivo, Agilent Technologies, Santa Clara, CA 95051, United States) with ESI (electrospray) ion source was used for the determination of mycotoxin concentrations of the samples. The mass spectrometer was operated in MRM (multiple reaction monitoring) scan mode and monitored two transitions (1 qualifier, 1 quantifier) of T-2 precursor ions in positive ion mode. The applied analytical method was validated for the LB medium. The correlation coefficient (R2) of the matrix-matched calibration was 0.9936, recovery from LB medium spiked with T-2 standard was 78 ± 13%, LOD (limit of detection) 3 µg/L and LOQ (limit of quantification) 11 µg/L.

### 5.4. Maintenance of Zebrafish and Egg Collection

Laboratory-bred wild type AB strain zebrafish were held in breeding groups of 30 females and 30 males at the Department of Aquaculture, Szent István University, Hungary, in a Tecniplast ZebTEC recirculation system (Tecniplast S.p.a., Buguggiate VA, Italy) at 25.5 °C ± 0.5 °C, pH 7.0 ± 0.2, conductivity 550 ± 50 µS (system water) and light/dark period of 14 h:10 h. Fish were fed twice a day with dry granulate food (Zebrafeed 400–600 µm, Sparos Lda., Olhão, Portugal) supplemented with freshly hatched live Artemia salina twice a week. Fish were placed in breeding tanks (Tecniplast S.p.a.) late in the afternoon the day before the experiment and allowed to spawn by removing the dividing walls the next morning. Spawning of individual pairs was delayed through time to allow a continuous supply of one-cell embryos.

### 5.5. Microinjection

Microinjection of zebrafish embryos (microinjector, capillary puller and parameters of capillary) was conducted as described by Csenki et al. [46]. Briefly, one-cell embryos were injected with different volumes: sphere diameter of 52 µm corresponded to an injection volume of 0.074 nL, 150 µm to 1.77 nL, 180 µm to 3.05 nL and 200 µm to 4.17 nL. These doses were used for each test solution (7 mg/L T-2, primary metabolites and degradation metabolites). After 2 h, coagulated and/or nonfertilized eggs were discarded and dividing eggs were transferred in groups of twenty into 6 cm diameter Petri dishes. Each treatment group contained 20 eggs in three replicates. Embryos were then incubated (Sanyo MIR-154) in system water at 26 °C ± 1 °C and a 14 h:10 h light/dark period and checked for lethal and sublethal effects under a microscope. System water was replaced every 24 h until 120 hpf. Digital images of embryos (72 hpf) and larvae (120 hpf) in lateral orientation were taken under a stereomicroscope at 30× magnification (Leica M205 FA, Leica DFC 7000T camera, Leica Application Suite X, Leica Microsystems GmbH, Wetzlar, Germany).

The qualification of strains was performed in three steps ((1) determination of mycotoxin toxicity baseline, (2) examination of bacterial metabolites toxicity, (3) identification of degradation product toxicity) so the results are comparable. By comparing the effects of normal metabolites and T-2 with the effects of degradation products, it can be determined which test substance caused the observed effects.

### 5.6. Toxicological Endpoints

Mortality values of injected embryos were determined at 72 and 120 hpf on the basis of egg coagulation, the lack of somite formation and the lack of heart function. Sublethal effects were examined at 72 and 120 hpf, the endpoints were hook-like tail, tail deformities, pericardial and yolk edema, yolk deformities, lens and head distortion, lack of hatching and lack of swim bladder. The frequency of deformities was compared to the number of living embryos at 72 and 120 h.

### 5.7. Statistics

Results were analyzed and graphs were plotted by GraphPad Prism 6.01 for Mac (GraphPad Software, San Diego, CA, USA). Data were checked for normality with the Shapiro–Wilk normality test. Significance differences were verified by Kruskal–Wallis analysis with Dunn’s multiple comparisons test. Results were compared to the results of noninjected control.

## Figures and Tables

**Table 1:** T-2 concentration of degradation samples detected by the analytical method (UHPLC-MS/MS). The supernatants and pellets of bacterial strains were evaluated separately. The initial T-2 concentration was 7 mg/L in all degradation experiments. Results of under detection limit are expressed as <LOD.

T-2 Concentration of Degradation Samples (mg/L)												
Supernatant						Pellet						
	A	B	C	Mean	SD			A	B	C	Mean	SD
**AK38**	5.81	5.49	4.94	5.42	0.44		**AK38**	2.53	1.55	1.11	1.73	0.73
**N774**	2.29	0.18	1.68	1.39	1.09		**N774**	<LOD	<LOD	<LOD	<LOD	<LOD
**N58**	<LOD	<LOD	<LOD	<LOD	<LOD		**N58**	<LOD	<LOD	<LOD	<LOD	<LOD
**NI2**	<LOD	<LOD	<LOD	<LOD	<LOD		**NI2**	<LOD	<LOD	<LOD	<LOD	<LOD
**NI1**	<LOD	<LOD	<LOD	<LOD	<LOD		**NI1**	<LOD	<LOD	<LOD	<LOD	<LOD
**N361**	6.61	6.48	5.78	6.29	0.45		**N361**	0.78	0.48	1.26	0.87	0.39
**NZS14**	0.03	2.43	1.89	1.45	1.26		**NZS14**	<LOD	<LOD	<LOD	<LOD	<LOD

## Supplementary Materials

The following are available online at https://www.mdpi.com/2072-6651/12/7/460/s1, Table S1: Effects of bacterial metabolites on the frequency of developmental deformities (x) in 72 and 120 hpf zebrafish embryos. Frequency are expressed as mean ± SD from three independent experiments in triplicate. Kruskal–Wallis followed by Dunn’s post hoc test was used. Values were compared to results of noninjected control (* *p* < 0.05, ** *p* < 0.01, *** *p* < 0.001). (td: tail deformities, pe: pericardial edema, ye: yolk edema, hd: head and lens distortion), Table S2: Effects of degradation metabolites and T-2 on the frequency of developmental deformities (x) in 72 and 120 hpf zebrafish embryos. Frequency are expressed as mean ± SD from three independent experiments in triplicate. Kruskal–Wallis followed by Dunn’s post hoc test was used. Values were compared to results of noninjected control (* *p* < 0.05, ** *p* < 0.01, *** *p* < 0.001). (td: tail deformities, pe: pericardial edema, ye: yolk edema, hd: head and lens distortion).

## References

[1] Marroquin-Cardona A., Johnson N., Phillips T.D., Hayes A.W. (2014). Mycotoxins in a changing global environment—A review. Food Chem. Toxicol..

[2] Streit E., Schatzmayr G., Tassis P., Tzika E., Marin D.E., Taranu I., Tabuc C., Nicolau A.I., Aprodu I., Puel O. (2012). Current Situation of Mycotoxin Contamination and Co-occurrence in Animal Feed—Focus on Europe. Toxins.

[3] Pestka J.J. (1994). Application of immunology to the analysis and toxicity assessment of mycotoxins. Food Agric. Immunol..

[4] Wu F. (2007). Measuring the economic impacts of Fusarium toxins in animal feeds. Anim. Feed. Sci. Technol..

[5] He J., Zhou T., Young J.C., Boland G.J., Scott P.M. (2010). Chemical and biological transformations for detoxification of trichothecene mycotoxins in human and animal food chains: A review. Trends Food Sci. Technol..

[6] Binder E.M. (2007). Managing the risk of mycotoxins in modern feed production. Anim. Feed. Sci. Technol..

[7] Fuchs E., Binder E.M., Heidler D., Krska R. (2002). Structural characterization of metabolites after the microbial degradation of type A trichothecenes by the bacterial strain BBSH 797. Food Addit. Contam..

[8] (2003). Mycotoxins: Risks in Plant, Animal, and Human Systems.

[9] Hsu I.-C., Smalley E.B., Strong F.M., Ribelin W.E. (1972). Identification of T-2 Toxin in Moldy Corn Associated with a Lethal Toxicosis in Dairy Cattle. Appl. Microbiol..

[10] Kalantari H., Moosavi M. (2010). Review on T-2 Toxin. Jundishapur J. Natl. Pharm. Prod..

[11] (2001). Opinion of the Scientific Committee on Food on Fusarium Toxins Part 5: T-2 Toxin and HT-2 Toxin.

[12] (1990). Selected Mycotoxins: Ochratoxins, Trichothecenes, Ergot.

[13] Boonchuvit B., Hamilton P.B., Burmeister H.R. (1975). Interaction of T-2 Toxin with Salmonella Infections of Chickens. Poult. Sci..

[14] Kanai K., Kondo E. (1984). Decreased Resistance to Mycobacterial Infection in Mice Fed a Trichothecene Compound (T-2 Toxin). Jpn. J. Med Sci. Biol..

[15] Yarom R. (1984). T-2 toxin effect on bacterial infection and leukocyte functions. Toxicol. Appl. Pharmacol..

[16] Jagadeesan V., Rukmini C., Vijayaraghavan M., Tulpule P. (1982). Immune studies with T-2 toxin: Effect of feeding and withdrawal in monkeys. Food Chem. Toxicol..

[17] Poston H.A., Coffin J.L., Combs G.F. (1982). Biological effects of dietary T-2 toxin on rainbow trout, Salmo gairdneri. Aquat. Toxicol..

[18] Yuan G., Wang Y.-M., Yuan X., Zhang T., Zhao J., Huang L., Peng S. (2014). T-2 toxin induces developmental toxicity and apoptosis in zebrafish embryos. J. Environ. Sci..

[19] Beardall J.M., Miller J.D., Miller J., Trenholm H. (1994). Diseases in Humans with Mycotoxins as Possible Causes. Mycotoxins in Grain: Compounds Other than Aflatoxin.

[20] Bhat R., Ramakrishna Y., Beedu S., Munshi K. (1989). Outbreak of Trichothecene Mycotoxicosis Associated with Consumption of Mould-Damaged Wheat Products in Kashmir Valley, India. Lancet.

[21] Joffe A.Z., Purchase I.F.H. (1974). Toxicity of Fusarium Poae and F. sporotrichioides and Its Relation to Alimentary Toxic Aleukia. Mycotoxins.

[22] Bata A. (1999). Detoxification of mycotoxin-contaminated food and feed by microorganisms. Trends Food Sci. Technol..

[23] Clements M.J., White D.G. (2004). Identifying Sources of Resistance to Aflatoxin and Fumonisin Contamination in Corn Grain. J. Toxicol. Toxin Rev..

[24] Munkvold G.P. (2003). Cultural Andgeneticapproaches Tomanagingmycotoxins Inmaize. Annu. Rev. Phytopathol..

[25] Sinha A., Sinha K. (1990). Insect pests, Aspergillus flavus and aflatoxin contamination in stored wheat: A survey at North Bihar (India). J. Stored Prod. Res..

[26] Champeil A., Fourbet J., Doré T., Rossignol L. (2004). Influence of cropping system on Fusarium head blight and mycotoxin levels in winter wheat. Crop. Prot..

[27] Dorner J.W., Cole R.J. (2002). Effect of application of nontoxigenic strains of Aspergillus flavus and A. parasiticus on subsequent aflatoxin contamination of peanuts in storage. J. Stored Prod. Res..

[28] Jard G., Liboz T., Mathieu F., Guyonvarc’H A., Lebrihi A. (2011). Review of mycotoxin reduction in food and feed: From prevention in the field to detoxification by adsorption or transformation. Food Addit. Contam. Part A.

[29] Guerre P. (2000). Interest of the treatments of raw materials and usage of adsorbents to decontaminate animal food containing mycotoxins. Rev. Méd. Vét..

[30] Kabak B., Dobson A.D.W., Var I. (2006). Strategies to Prevent Mycotoxin Contamination of Food and Animal Feed: A Review. Crit. Rev. Food Sci. Nutr..

[31] Murphy P.A., Rice J.L.G., Ross P.F. (1993). Fumonisin B1, B2 and B3 content of Iowa, Wiscinsin, Illinois corn and screenings. J. Agric. Food Chem..

[32] Burrows E.P., Szafraniec L.L. (1987). Hypochlorite-Promoted Transformations of Trichothecenes, 3. Deoxynivalenol. J. Nat. Prod..

[33] Freimund S., Sauter M., Rys P. (2003). Efficient Adsorption of the Mycotoxins Zearalenone and T?2 Toxin on a Modified Yeast Glucan. J. Environ. Sci. Health Part B.

[34] Dvorska J.E., Pappas A.C., Karadas F., Speake B.K., Surai P.F. (2007). Protective effect of modified glucomannans and organic selenium against antioxidant depletion in the chicken liver due to T-2 toxin-contaminated feed consumption. Comp. Biochem. Physiol. Part C Toxicol. Pharmacol..

[35] Kubena L.F., Edrington T.S., Harvey R., Buckley S., Phillips T.D., Rottinghaus G., Casper H.H. (1997). Individual and combined effects of fumonisin B1 present in Fusarium moniliforme culture material and T-2 toxin or deoxynivalenol in broiler chicks. Poult. Sci..

[36] Raju M., Devegowda G. (2000). Influence of esterified-glucomannan on performance and organ morphology, serum biochemistry and haematology in broilers exposed to individual and combined mycotoxicosis (aflatoxin, ochratoxin and T-2 toxin). Br. Poult. Sci..

[37] Hathout A., Aly S. (2014). Biological detoxification of mycotoxins: A review. Ann. Microbiol..

[38] Cserhati M., Kriszt B., Krifaton C., Szoboszlay S., Hahn J., Toth S., Nagy I., Kukolya J. (2013). Mycotoxin-degradation profile of Rhodococcus strains. Int. J. Food Microbiol..

[39] Beeton S., Bull A.T. (1989). Biotransformation and detoxification of T-2 toxin by soil and freshwater bacteria. Appl. Environ. Microbiol..

[40] Ueno Y., Nakayama K., Ishii K., Tashiro F., Minoda Y., Omori T., Komagata K. (1983). Metabolism of T-2 toxin in *Curtobacterium* sp. strain 114-2. Appl. Environ. Microbiol..

[41] Kuca K., Dohnal V., Jezkova A., Jun D. (2008). Metabolic pathways of T-2 toxin. Curr. Drug Metab..

[42] Schuhmacher-Wolz U., Heine K., Schneider K. (2010). Report on toxicity data on trichothecene mycotoxins HT-2 and T-2 toxins. EFSA Support. Publ..

[43] Wu Q., Dohnal V., Huang L., Ramalho T.C., Yuan Z. (2010). Metabolic pathways of trichothecenes. Drug Metab. Rev..

[44] Boudergue C., Burel C., Dragacci S., Favrot M., Fremy J., Massimi C., Prigent P., Debongnie P., Pussemier L., Boudra H. (2009). Review of mycotoxin-detoxifying agents used as feed additives: Mode of action, efficacy and feed/food safety. EFSA Support. Publ..

[45] European Food Safety Authority (2010). Statement on the establishment of guidelines for the assessment of additives from the functional group ‘substances for reduction of the contamination of feed by mycotoxins’. EFSA J..

[46] Csenki-Bakos Z., Garai E., Risa A., Cserháti M., Bakos K., Márton D., Bokor Z., Kriszt B., Urbányi B. (2019). Biological evaluation of microbial toxin degradation by microinjected zebrafish (Danio rerio) embryos. Chemosphere.

[47] Schubert S., Keddig N., Hanel R., Kammann U. (2014). Microinjection into zebrafish embryos (Danio rerio)—A useful tool in aquatic toxicity testing?. Environ. Sci. Eur..

[48] Adhikari M., Negi B., Kaushik N., Adhikari A., Al-Khedhairy A., Kaushik N.K., Choi E.H. (2017). T-2 mycotoxin: Toxicological effects and decontamination strategies. Oncotarget.

[49] Ueno Y. (1984). Toxicological features of T-2 toxin and related trichothecenes. Fundam. Appl. Toxicol..

[50] Chan P.-C., Gentry P. (1984). LD50 values and serum biochemical changes induced by T-2 toxin in rats and rabbits. Toxicol. Appl. Pharmacol..

[51] Chi M.S., Mirocha C.J., Kurtz H.J., Weaver G., Bates F., Shimoda W., Burmeister H.R. (1977). Acute Toxicity of T-2 Toxin in Broiler Chicks and Laying Hens. Poult. Sci..

[52] Ueno Y. (1980). Trichothecene Mycotoxins Mycology, Chemistry, and Toxicology. Advances in Nutritional Research.

[53] Jewers K., Saveur B. (1990). Mycotoxins and Their Effect on Poultry Production. L’aviculture en Méditerranée.

[54] Devreese M., De Backer P., Croubels S. (2013). Different methods to counteract mycotoxin production and its impact on animal health. Vlaams Diergeneeskundig Tijdschrift.

[55] Doi K., Ishigami N., Sehata S. (2008). T-2 Toxin-induced Toxicity in Pregnant Mice and Rats. Int. J. Mol. Sci..

[56] Wyatt R.D., Weeks B.A., Hamilton P.B., Burmeister H.R. (1972). Severe Oral Lesions in Chickens Caused by Ingestion of Dietary Fusariotoxin T-21. Appl. Microbiol..

[57] Cheeke P.R. (1995). Endogenous toxins and mycotoxins in forage grasses and their effects on livestock. J. Anim. Sci..

[58] Háhn J., Szoboszlay S., Tóth G., Kriszt B. (2017). Assessment of bacterial biodetoxification of herbicide atrazine using Aliivibrio fischeri cytotoxicity assay with prolonged contact time. Ecotoxicology.

[59] Rachitha P., Khanum F. (2014). T-2 mycotoxin induced toxicity: A review. Int. J. Curr. Res..

[60] Park J., Lee H.-H., Youn K., Kim S., Jung B., Lee J., Seo Y.-S. (2014). Transcriptome analyses to understand effects of the Fusarium deoxynivalenol and nivalenol mycotoxins on Escherichia coli. J. Biotechnol..

