# Supplementary material for: Qualifying the T-2 Toxin-Degrading Properties of Seven Microbes with Zebrafish Embryo Microinjection Method
Source: Toxins (Basel). 2020 Jul 18;12(7):460. doi: 10.3390/toxins12070460 (PMC7405011; doi:10.3390/toxins12070460)
Supplement: Supplementary file 1 [file toxins-12-00460-s001.pdf]

Qualifying the T-2 Toxin-Degrading Properties of Seven Microbes with Zebrafish Embryo Microinjection Method

Table S1. Effects of bacterial metabolites on the frequency of developmental deformities (x) in 72 and 120 hpf zebrafish embryos. Frequency are expressed as mean ± SD from three independent experiments in triplicate. Kruskal–Wallis followed by Dunn's post hoc test was used. Values were compared to results of noninjected control (* $p < 0.05$, ** $p < 0.01$, *** $p < 0.001$). (td: tail deformities, pe: pericardial edema, ye: yolk edema, hd: head and lens distortion).

Bacterial Metabolites											
		72 hpf					120 hpf				
		Deformation Type				Freq ±SD (%)	Deformation Type				Freq ±SD (%)
**Strains**	**nL**	**td**	**pe**	**ye**	**hd**		**td**	**pe**	**ye**	**hd**	
**AK38**	0.074	x	-	-	-	2.24 ± 1.06	x	-	-	-	8.91 ± 7.82
	1.77	x	-	-	-	5.4 ± 1.94	x	-	-	-	6.87 ± 9.69
	3.05	x	-	-	-	7.29 ± 0.94	x	-	-	-	7.66 ± 5.56
	4.17	x	-	-	-	11.95 ± 2.19	x	-	-	-	12.76 ± 3.26
**N774**	0.074	x	-	-	-	1.27 ± 1.15	-	-	x	-	3.17 ± 1.12
	1.77	x	-	-	-	4.38 ± 1.12	-	-	x	-	5.34 ± 1.42
	3.05	x	-	-	-	7.06 ± 1.08	-	-	x	-	7.7 ± 1.34
	4.17	x	-	-	-	13.14 ± 3.05 *	-	-	x	-	16.06 ± 2.48 *
**N58**	0.074	x	x	x	x	16.06 ± 0.72	x	x	x	x	16.43 ± 1.13
	1.77	x	x	x	x	33.35 ± 8.29	x	x	x	x	38.46 ± 3.45
	3.05	x	x	x	x	56.02 ± 4.57	x	x	x	x	57.32 ± 0.73
	4.17	x	x	x	x	82.03 ± 4.07	x	x	x	x	84.13 ± 2.42
**NI2**	0.074	x	-	x	-	15.1 ± 7.26	x	-	x	-	16.43 ± 3.17
	1.77	x	-	x	-	46.24 ± 5.39	x	-	x	-	48.31 ± 5.48
	3.05	x	-	x	-	92.08 ± 8.54 **	x	-	x	-	100.0 ± 0.00 **
	4.17	x	-	x	-	100.0 ± 0.00 ***	x	-	x	-	100.0 ± 0.00 ***
**NI1**	0.074	-	-	-	-	0.97 ± 0.84	-	-	-	-	2.75 ± 0.65
	1.77	-	-	-	-	1.48 ± 0.55	-	-	-	-	2.46 ± 1.56
	3.05	-	-	-	-	2.61 ± 0.82	-	-	-	-	1.73 ± 1.05
	4.17	-	-	-	-	1.66 ± 1.49	-	-	-	-	2.13 ± 1.29
**N361**	0.074	-	x	x	-	2.71 ± 0.39	x	x	x	-	3.63 ± 1.17
	1.77	-	x	x	-	6.26 ± 1.16	x	x	x	-	6.52 ± 1.37
	3.05	-	x	x	-	8.24 ± 1.68	x	x	x	-	9.91 ± 1.15
	4.17	-	x	x	-	10.52 ± 2.19	x	x	x	-	13.48 ± 2.48
**NZS14**	0.074	x	x	x	-	3.49 ± 0.46	x	x	x	-	6.82 ± 2.11
	1.77	x	x	x	-	5.97 ± 0.83	x	x	x	-	8.04 ± 1.21
	3.05	x	x	x	-	10.98 ± 0.77	x	x	x	-	12.2 ± 1.34
	4.17	x	x	x	-	24.82 ± 5.32 *	x	x	x	-	28.17 ± 3.13 *
**Non-inj-c**	-	-	-	-	-	0.00 ± 0.00	-	-	-	-	0.00 ± 0.00

Table S2. Effects of degradation metabolites and T-2 on the frequency of developmental deformities (x) in 72 and 120 hpf zebrafish embryos. Frequency are expressed as mean ± SD from three independent experiments in triplicate. Kruskal–Wallis followed by Dunn's post hoc test was used. Values were compared to results of noninjected control (* $p < 0.05$, ** $p < 0.01$, *** $p < 0.001$). (td: tail deformities, pe: pericardial edema, ye: yolk edema, hd: head and lens distortion).

Degradation Products											
		72 hpf					120 hpf				
		Deformation Type					Deformation Type				
**Strains**	**nL**	**td**	**pe**	**ye**	**hd**	**Freq ±SD (%)**	**td**	**pe**	**ye**	**hd**	**Freq ±SD (%)**
**AK38**	0.074	x	-	-	-	3.75 ± 1.77	x	-	-	-	4.24 ± 1.06
	1.77	x	-	-	-	8.42 ± 1.31	x	-	-	-	8.54 ± 1.94
	3.05	x	-	-	-	11.23 ± 2.01 *	x	-	-	-	12.72 ± 0.94
	4.17	x	-	-	-	27.1 ± 3.58 *	x	-	-	-	8.95 ± 3.79
**N774**	0.074	x	x	x	-	17.01 ± 5.32	x	x	x	-	17.31 ± 8.32
	1.77	x	x	x	-	33.34 ± 3.5	x	x	x	-	48.32 ± 7.21
	3.05	x	x	x	-	53.06 ± 5.75 *	x	x	x	-	53.06 ± 5.75
	4.17	x	x	x	-	75.97 ± 4.5 **	x	x	x	-	75.97 ± 4.5
**N58**	0.074	x	x	x	x	1.7 ± 1.39	x	x	x	x	16.06 ± 0.72
	1.77	x	x	x	x	13.08 ± 4.99	x	x	x	x	33.35 ± 8.29
	3.05	x	x	x	x	45.33 ± 5.18	x	x	x	x	56.02 ± 4.57
	4.17	x	x	x	x	59.2 ± 8.2 **	x	x	x	x	82.03 ± 4.07 **
**NI2**	0.074	x	-	-	-	8.29 ± 0.73	x	-	-	-	15.1 ± 7.26
	1.77	x	-	-	-	19.73 ± 1.99	x	-	-	-	66.24 ± 1.16
	3.05	x	-	-	-	32.87 ± 3.93	x	-	-	-	92.08 ± 8.54
	4.17	x	-	-	-	52.5 ± 3.54 *	x	-	-	-	100.0 ± 0.00 *
**NI1**	0.074	-	-	-	-	0.86 ± 1.00	-	-	-	-	0.97 ± 0.84
	1.77	-	-	-	-	1.14 ± 0.87	-	-	-	-	2.49 ± 0.55
	3.05	-	-	-	-	1.61 ± 1.47	-	-	-	-	5.61 ± 0.82
	4.17	-	-	-	-	2.33 ± 1.34	-	-	-	-	6.66 ± 1.49
**N361**	0.074	x	x	x	-	3.78 ± 1.37	x	-	-	-	3.78 ± 1.37
	1.77	x	x	x	-	15.61 ± 3.64	x	-	-	-	15.61 ± 3.64
	3.05	x	x	x	-	50.29 ± 2.61 *	x	-	-	-	53.97 ± 1.07 *
	4.17	x	x	x	-	84.57 ± 10.63 **	x	-	-	-	88.07 ± 8.31 **
**NZS14**	0.074	x	x	x	-	3.76 ± 1.48	x	-	-	x	3.89 ± 1.51
	1.77	x	x	x	-	29.1 ± 2.53	x	-	-	x	29.42 ± 0.83
	3.05	x	x	x	-	48.69 ± 3.09	x	-	-	x	48.91 ± 0.77
	4.17	x	x	x	-	81.00 ± 7.64 *	x	-	-	x	81.34 ± 5.32 *
**T-2**	0.074	x	x	x	x	10.52 ± 4.72	x	x	x	x	15.43 ± 9.76
	1.77	x	x	x	x	29.82 ± 9.89	x	x	x	x	30.4 ± 4.9
	3.05	x	x	x	x	88.31 ± 4.74 *	x	x	x	x	85.11 ± 9.6 *
	4.17	x	x	x	x	100.00 ± 0.00 **	x	x	x	x	100.00 ± 0.00 **
